# The Inhibitory Effect of Curosurf^®^ and Alveofact^®^ on the Formation of Neutrophil Extracellular Traps

**DOI:** 10.3389/fimmu.2020.582895

**Published:** 2021-01-19

**Authors:** Annabell Schulz, Laia Pagerols Raluy, Jan Philipp Kolman, Ingo Königs, Magdalena Trochimiuk, Birgit Appl, Konrad Reinshagen, Michael Boettcher, Julian Trah

**Affiliations:** Department of Pediatric Surgery, University Medical Center Hamburg-Eppendorf (UKE), Hamburg, Germany

**Keywords:** neutrophil granulocytes, neutrophil extracellular traps, pulmonary surfactant, anti-inflammatory, alveofact^®^, curosurf^®^

## Abstract

**Background:**

Neutrophil extracellular traps (NETs) are a defense mechanism in which neutrophils cast a net-like structure in response to microbial infection. NETs consist of decondensed chromatin and about 30 enzymes and peptides. Some components, such as neutrophil elastase (NE) and myeloperoxidase (MPO), present antimicrobial but also cytotoxic properties, leading to tissue injury. Many inflammatory diseases are associated with NETs, and their final role has not been identified. Pulmonary surfactant is known to have immunoregulatory abilities that alter the function of adaptive and innate immune cells. The aim of this study was to investigate the hypothesis that natural surfactant preparations inhibit the formation of NETs.

**Methods:**

The effect of two natural surfactants (Alveofact^®^ and Curosurf^®^) on spontaneous and phorbol-12-myristate-13-acetate–induced NET formation by neutrophils isolated by magnetic cell sorting from healthy individuals was examined. NETs were quantitatively detected by absorption and fluorometric-based assays for the NET-specific proteins (NE, MPO) and cell-free DNA. Immunofluorescence microscopy images were used for visualization.

**Results:**

Both surfactant preparations exerted a dose-dependent inhibitory effect on NET formation. Samples treated with higher concentrations and with 30 min pre-incubation prior to stimulation with phorbol-12-myristate-13-acetate had significantly lower levels of NET-specific proteins and cell-free DNA compared to untreated samples. Immunofluorescence microscopy confirmed these findings.

**Conclusions:**

The described dose-dependent modulation of NET formation ex vivo suggests an interaction between exogenous surfactant supplementation and neutrophil granulocytes. The immunoregulatory effects of surfactant preparations should be considered for further examination of inflammatory diseases.

## Introduction

Neutrophil granulocytes play a major role in defending the lung against invading pathogens (1). As part of the innate immune system, they reach the site of infection as one of the first immune cells to attack microorganisms by using defense mechanisms such as phagocytosis and degranulation (2). A particular form of host defense performed by these cells is neutrophil extracellular traps (NETs). To form NETs, neutrophils undergo a specific form of programmed cell death, termed NETosis, which leads to the release of web-like decondensed DNA and antimicrobial granular proteins into the extracellular space (3). Besides the beneficial effect of entrapping infectious agents, an inordinate amount of NETs leads to tissue injury due to their cytotoxic components such as histones, neutrophil elastase (NE), myeloperoxidase (MPO), and other neutrophil proteases, thus contributing to many inflammatory lung diseases (4–6). NETosis may be triggered by a variety of stimuli such as bacteria, viruses, fungi, and protozoa, among others. In vitro NETosis can be elicited through treatment with phorbol 12-myristate 13-acetate (PMA) or interleukin (IL)-8 (1, 7–10). Due to the involvement of NETs in many inflammatory and chronic lung diseases, they might serve as therapeutic targets, however this has not been explored in detail so far (4, 11, 12).

Pulmonary surfactant is a composition of mostly phospholipids (80%), cholesterol (10%), and the four surfactant proteins (SP): SP-A, SP-B, SP-C, and SP-D (10%). It is secreted by alveolar type II cells, lining the alveolar epithelium and thereby reducing the surface tension at the air–liquid interface. This function is important for ensuring the compliance of the lung, supporting gas exchange, and preventing alveolar collapse during exhalation (13). Consequently, a lack of surfactant leads to respiratory failure in premature infants, causing respiratory distress syndrome, which requires mechanical ventilation and exogenous surfactant replacement (14). In Germany, there are two typical clinically used animal-derived (natural) surfactants. Bovine-derived Alveofact^®^ and porcine-derived Curosurf^®^. Both consist of multiple components and differ significantly in composition. While Alveofact^®^ holds a larger portion of both SP-B and SP-C, Curosurf^®^ contains more Dipalmitoylphosphatidylcholine, a major phospholipid in surfactant (15). Recommended dosage of Alveofact^®^ with 50–100 mg/kg differs from recommendations for Curosurf^®^ with 100–200 mg/kg. However, the cumulative dosage of both surfactants may differ from the recommendations due to clinically necessary repeated applications (16, 17). In terms of *in vivo* effectiveness, animal-derived (natural) surfactants containing SP-B and SP-C have proven to be superior to synthetic animal protein-free surfactants (18). Apart from the commonly known biophysical functions, the role of pulmonary surfactant in the innate immune system has been elucidated in the last two decades (19). The immunoregulatory properties are primarily exerted by the surfactant proteins, especially SP-A and SP-D. These hydrophilic proteins belong to the collectin family and are able to interact with a variety of pathogens and other targets (neutrophils, surfactant phospholipids, and DNA) *via* a carbohydrate recognition domain (CRD; lectin domain) and further collectin receptors (20). They are not only able to opsonize pathogens *via* their CRDs and thus enhance phagocytosis by immune cells, but can also modulate and even inhibit the functions of these cells through anti-inflammatory signals (21). For example, SP-A and SP-D deficiency renders mice incapable of terminating neutrophilic inflammation (22, 23). Moreover, SP-D can diminish lipopolysaccharide (LPS)-induced NET formation (24). The hydrophobic surfactant proteins SP-B and SP-C play a crucial role in reducing surface tension (13, 25). However, more recent studies find that they and other surfactant phospholipids also contribute to immunomodulation (26–29).

In the present study, the effect of two common clinically used surfactants (Alveofact^®^, Curosurf^®^) on spontaneous and PMA-induced NET formation was investigated. Alveofact^®^ and Curosurf^®^ contain exclusively SP-B and SP-C, along with phospholipids, but no SP-A or SP-D that have been proven to be effective against neutrophilic inflammation and NET formation (23, 24, 30). We were able to assess the immunoregulatory effects of these natural surfactants on NET formation in neutrophil granulocytes.

## Materials and Methods

### Surfactant Preparations and Chemokines

Two natural surfactant preparations common in clinical use were examined: Curosurf^®^ (Chiesi, Hamburg, Germany) is prepared from minced porcine lungs and supplied as a ready-to-use intratracheal suspension. Alveofact^®^ (Lyomark Pharma, Oberhaching, Germany) is a surfactant isolated from bovine lung lavage and prepared as a lyophilized powder that has to be reconstituted with the provided solvent prior to use. The surfactants were used and stored according to the manufacturers’ recommendations. To obtain approximately similar concentrations *in vitro* we covered a range of concentrations from 0.001–2.5 mg/ml.

Moreover, phorbol-12-myristate-13-acetate (PMA), a common chemical stimulator for NETosis (10), from Cayman Chemical (USA) was used.

### Isolation of Neutrophils

Blood was obtained with informed, signed consent from healthy local donors following approval by the Ethics Committee of the Hamburg Medical Association (PV5921). The blood was collected in EDTA-K monovettes (Sarstedt, Nümbrecht, Germany). Neutrophil granulocytes were isolated using the MACSxpress^®^ Whole Blood Neutrophil Isolation Kit according to the manufacturer’s protocol (Miltenyi Biotec, Bergisch Gladbach, Germany). Briefly: 8ml of whole blood was mixed with 4ml MACSxpress^®^ Isolation Mix, incubated for 5 min at room temperature (RT) in the MACSmix™ Tube Rotator at 12 rpm, and magnetically separated with the MACSxpress^®^ Separator for 15 min. After centrifugation of the target cell fraction at 300 × g for 10 min at 4°C, residual erythrocytes were lysed at 4°C by resuspending the cell pellet in 2 ml of H_2_O for 30 s prior to adding 0.7 ml of 0.6 M KCl (Sigma-Aldrich, Saint Louis, MO, USA). Neutrophils were washed with phosphate-buffered saline and resuspended in RPMI 1640 (Thermo Fischer, Waltham, MA, USA) containing 1% bovine serum albumin (Sigma-Aldrich, Saint Louis, MO, USA).

Purity of the extracted neutrophils (>95%) was assured *via* fluorescence-activated cell sorting (FACS) using anti-CD15-FITC (mAb HI98, IgM) and anti-CD16-PerCP (mAb 3G8, IgG1) antibodies (BioLegend, San Diego, CA, USA). Cell morphology was analyzed by hematoxylin and eosin staining.

### Cell Treatments

Neutrophils were resuspended in Gibco^®^ RPMI 1640 media containing 1% bovine serum albumin (BSA) for the MPO assay and in Gibco ^®^ RPMI 1640 media containing 1% BSA and 0.1% calcium chloride for the NE and cell-free DNA (cfDNA) assay. For the MPO assay, the cells were seeded into a 96-well cell culture plate at a density of 2×10^5^ cells per well. Neutrophils undergoing NE and cfDNA assay were seeded in a 24-well cell culture plate at a concentration of 1×10^6^ cells/well. After 2-h incubation at 37°C, the cell culture plates were treated with Curosurf^®^ (0.001, 0.1, 1 mg/ml) or Alveofact^®^ (0.025, 0.25, 2.5 mg/ml) at different PMA incubation time points to investigate a possible preventive and/or therapeutic effect on NETosis. The cells were treated 30 min prior, simultaneously, and 10 min after stimulation with 100 nM PMA per well. After 3-h incubation at 37°C, NETosis assays were performed.

### Assays for Measurement of Neutrophil Extracellular Trap Formation

To quantify the amount of NETs, three different assays that measured NET-specific proteins and cfDNA were performed. The neutrophil MPO activity assay kit (MPO) and NETosis assay kit for measuring NET-derived MPO and NE, respectively from Cayman Chemicals were used according to the manufacturer’s instructions. The cfDNA assay was performed following the protocol of Fuchs et al. (31). To measure the effect of timing and dosage, we investigated three treatment time points (30 min before, simultaneously, and 10 min after activation with PMA) and three concentrations of Curosurf^®^ (1 µg/ml, 100 µg/ml, 1 mg/ml) and Alveofact^®^ (25 µg/ml, 250 µg/ml, 2.5 mg/ml). Untreated and unactivated cells were used as the negative control; untreated but activated cells served as the positive control.

For the MPO assay, cells were seeded and treated in a 96-well plate. Then, the plate was centrifuged at 1200 rpm for 10 min at RT, followed by transfer of the supernatants into a new plate. After adding 50 µl 3,3′,5,5′-tetramethylbenzidine (TMB), the absorbance of the supernatants was measured at 375 nm. The measured absorbance was proportional to the amount of MPO in the sample. Using the NETosis assay kit, only NE and DNA bound to the NETs were measured as the supernatant was washed out twice after treatment, leaving only adhered NETs on the bottom of the well. S7 nuclease was added to cleave the NETs and release NE and DNA into the supernatant, which could be used for both the NE assay and cfDNA assay.

For the NE assay, the supernatant was transferred into a 96-well plate and incubated with 100 µl 500 nM *N*-methoxysuccinyl-Ala-Ala-Pro-Val *p*-nitroanilide for 2 h at 37°C before the absorbance was read at 405 nm.

Cell-free DNA was detected by mixing 50 µl 1:2 diluted supernatant with 50 µl SYTOX Orange or 50 µl dilution buffer (0.1% BSA and 2 mM EDTA in DPBS). Fluorescence measurement was performed immediately afterwards at excitation, 544 nm; emission, 590 nm; cut off, 570 nm.

### Enzyme-Linked Immunosorbent Assay

The concentration of cytosolic PAD4 in neutrophils was measured using the Human PADI4 ELISA kit (MyBioSource, San Diego, USA) according to the manufacturer’s instructions. Protein extraction was carried out using RIPA Buffer with cOmplete^™^ Mini Protease Inhibitor Cocktail (Merck KGaA, Darmstadt, Germany). The protein levels were estimated from a generated standard curve. The optical densities were examined at a 450-nm wavelength using a microplate reader (Molecular Devices, Sunnyvale, USA).

### Immunostaining and Imaging

Neutrophils (2×10^5^/well) were seeded into 12-well plates containing coverslips and incubated for 2 h at 37°C. Medium was removed and replaced with or without surfactant solution 30 min before, simultaneously, and 10 min after stimulation with 100 nM PMA per well. The surfactant concentrations were analogous to that of the other assays (Curosurf^®^, 0.001, 0.1, and 1 mg/ml; Alveofact^®^, 0.025, 0.25, 2.5 mg/ml). After 3-h incubation at 37°C, the cells were washed twice with DPBS and fixed overnight at -20°C with 99% methanol. After fixation, the cells were immunostained. Cells were washed twice with DPBS, blocked with 1% BSA–DPBS for 1 h at RT, and incubated with either primary specific or isocontrol antibody solution for 1 h at RT. The following antibodies were used: 1:200 anti-NE antibody (Abcam, UK), 1:100 anti-MPO antibody (Abcam) and anti–citrullinated histone H3 R2+R8+R17 (H3cit) antibody (Abcam). Subsequently, the cells were incubated with secondary antibodies for 1 h at RT after washing twice with DPBS. NE and H3cit detection were assessed with an anti-rabbit secondary antibody conjugated with Alexa Fluor 647 (1:200, Abcam), MPO was detected with FITC-conjugated anti-mouse secondary antibody (1:1000, Abcam). After a three-wash step, the cells were counterstained with 1:1000 4′,6-diamino-2-phenylindole (DAPI) and mounted with Fluoromount-G (SouthernBiotech, USA). Microscopy images were taken using Apotome.2 by Zeiss (Carl Zeiss AG, Oberkochen, Germany) at ×40 magnification and processed using ImageJ software (Version 1.51, NIH, USA).

### Flow Cytometry

A total of 1x10^6^ neutrophils were treated with indicated doses at different timepoints, supernatant was collected and adherent cells were detached by incubation with Accutase (Capricorn Scientific, Ebsdorfergrund, Germany) for 30–60 min. Cells were washed twice with PBS (Thermo Fischer, Waltham, MA, USA) and labeled with propidium iodide (PI) and Annexin-V-FITC (Becton, Dickinson and Company, Franklin Lakes, NJ, USA) or anti-CD11b-VioBlue (mAb REA713, IgG1) and anti-CD66b-APC (mAb REA306, IgG1) antibodies (Miltenyi Biotec, Bergisch Gladbach, Germany) or anti-P2Y6 Receptor-FITC (Alomone Labs, Jerusalem, Israel) as described in the manufacturer’s protocols. Analysis was performed with a flow cytometer (FACSCanto™ II, Becton, Dickinson, and Company, Franklin Lakes, NJ, USA). Data were analyzed using BD FACSDiva™ (Becton, Dickinson and Company, Franklin Lakes, NJ, USA).

### Determination of Intracellular Levels of Reactive Oxygen Species

The intracellular ROS levels were determined as described previously by Balaiya et al. using the oxidation-sensitive fluorescent DHR-123 (ThermoFisher Scientific, Massachusetts, USA) (32, 33). Cells were seeded at a density of 0,5x10^5^ into black/flat clear bottom 96-well plates (Corning Incorporated, New York, NY, USA), treated as described and loaded with 5 μM DHR-123. Cells were then incubated for 30 min in the dark at culture condition and 30 min on the plate shaker at 35 rpm at RT. Fluorescence was measured at excitation/emission =505/534 nm using a microplate reader (Flex Station^®^ 3, Molecular Devices, San Jose, CA, USA).

### Intracellular Calcium Mobilization

To detect intracellular calcium mobilization, the Fluo-8 No Wash Calcium Assay kit was used, following manufacturer’s protocol (ab112129, Abcam, Cambridge, Great Britain). Briefly, 1x10^5^ cells per well were seeded in black/flat clear bottom 96-well plates (Corning Incorporated, New York, NY, USA) and settled at 37°C with 5% CO**_2_**. Cells were treated as described before. Fluo-8 stock solution was added together with PMA stimulation. Cells were incubated for 30 min at culture condition (37°C, 5% CO_2_) and afterwards 30 min at room temperature. Fluorescence was measured at excitation/emission = 490/525nm continuously every 15 min for 2 h using a microplate reader (Flex Station^®^ 3). Peak results for Calcium influx at 2 h after stimulation are displayed as normalized data on PMA-stimulated controls.

### Statistical Analysis

Each assay was performed in three biological replicates (n = 3). All data were analyzed with SPSS Statistics 26 (IBM, Ehningen, Germany) and GraphPad Prism 8 (San Diego, USA). Differences between groups were calculated using ANOVA with Dunnett’s correction for repeated measurements. Data are presented as mean ± SD of values from three independent experiments. Significance level was set as p<0.05 (*<0.05, **<0.005, ***<0.0005, ****<0.0001).

## ResuLts

### Curosurf^®^ and Alveofact^®^ Generate No Pro-Inflammatory Effect

**Figure 1:**
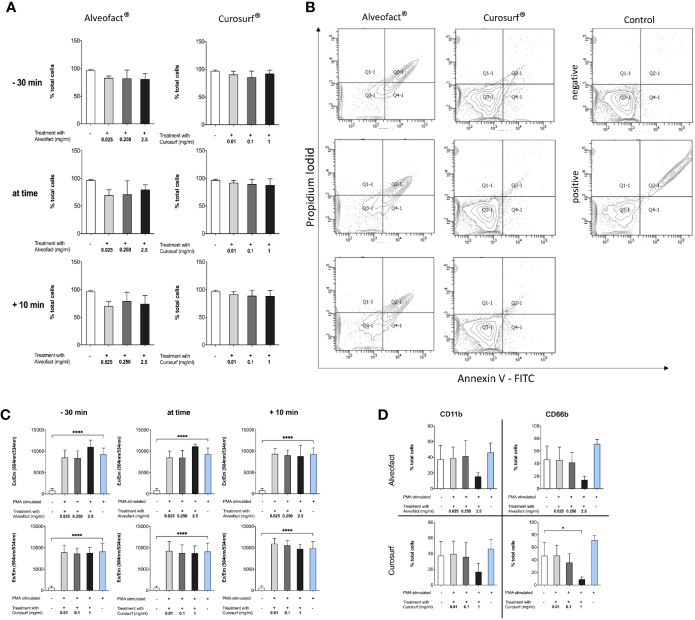
Neutrophils are not altered by animal derived surfactants. **(A**, **B)** Treatment with surfactant does not change survival of neutrophils. **(A)** Cells were treated and stained with PI and Annexin-V. Double negative cells are counted as living cells and displayed as mean+-SD of three independent experiments with no significant changes between untreated and unstimulated cells versus cells treated with surfactant at different timepoints and different doses. **(B)** Representative blot of 30 min pretreatment with surfactant. **(C)** Production of reactive oxygen species measured with Dihydrorhodamin-123. Treatment with surfactant does not alter the ability of neutrophils to produce ROS. **(D)** Neutrophils show no change in activation compared to untreated control. Figure shows 30 min pretreatment and is representative for all other timepoints. Only maximum dose of Curosurf^®^ shows a decrease of the activation marker CD66b. Significance level was set as p<0.05 (*<0.05, **<0.005, ***<0.0005, ****<0.0001).

Neither Curosurf^®^ nor Alveofact^®^ cause activation in neutrophils as indicated by staining with CD11b and CD66b antibodies (**Figure 1D**). Furthermore, they do not induce apoptosis or necrosis in neutrophils (**Figures 1A, B**) with a consistent ability of neutrophils to generate reactive oxygen species (**Figure 1C**). Also, we could not find a significant release of NETs in the NE, MPO or cfDNA assay either. (data not shown). This was tested by incubating the cells with the same dosages of Curosurf^®^ and Alveofact^®^ in the absence of PMA as a stimulator for NETosis. Compared to spontaneous NET formation, higher levels of MPO, NE, or cfDNA were not detectable.

### Curosurf^®^

**Figure 2:**
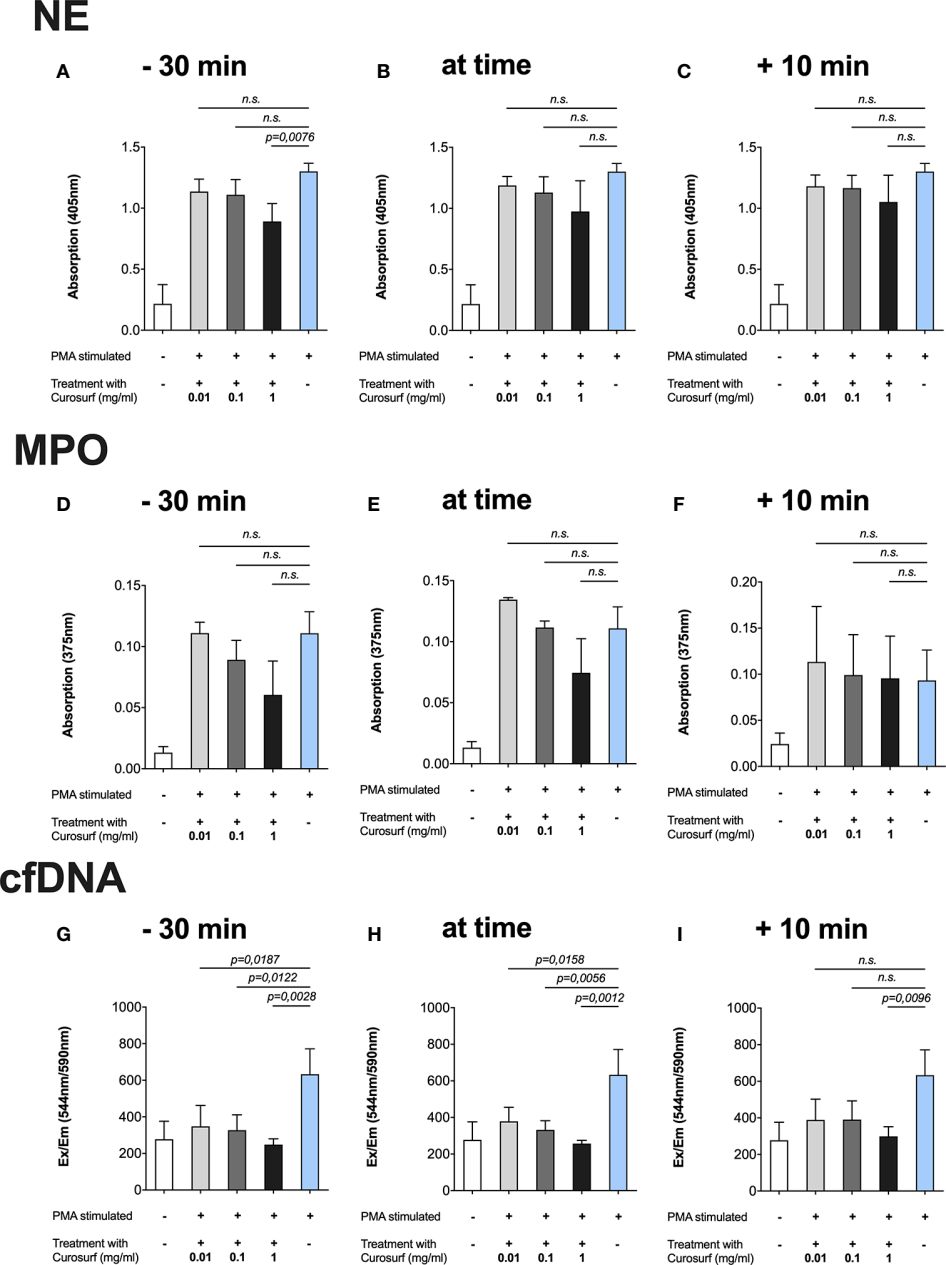
Curosurf^®^ reduces PMA-induced formation of NETs. Neutrophils were incubated with Curosurf^®^ at three different time points in relation to activation with 100 nm PMA, and then afterwards the NET-specific components were measured in three different assays. Cells that were untreated but stimulated with 100 nm PMA served as positive control in each assay. **(A**–**C)** There was a trend toward a dose- and time-dependent decrease in NE, but only the highest dose of 1 mg/ml paired with the longest incubation time of 30 min showed a significant result. **(D**–**F)** Proportionate intensity of the blue color emitted by TMB as a chromogenic substrate represented the amount of MPO and showed a significant decrease of NETs for the highest dose at each time point. **(G**–**I)** SYTOX Orange assessment of the amount of cfDNA. Significantly less cfDNA was observed in almost every variant of time and dose.

The highest concentration of Curosurf^®^ (1 mg/ml) paired with the longest incubation time, i.e., 30 min prior to activation with PMA, significantly reduced NE (p = 0.0076), MPO (p = 0.0027) and cfDNA (p = 0.0026) (**Figures 2A, D, G**). In the simultaneous activation/treatment and 10-min treatment after activation groups, samples treated with 1 mg/ml of Curosurf^®^ had significantly decreased MPO (**Figures 2E, F**) and cfDNA (**Figures 2H, I**), whereas NE (**Figures 2B, C**) exhibited no significant changes.

Similarly, only cfDNA was significantly decreased after treatment with lower concentrations of Curosurf^®^ (1 µg/ml, 100 µg/ml) and treatment simultaneously or 30 min prior to activation (**Figures 2G, H**). Although no statistical significance was observed for NE and MPO following treatment with lower Curosurf^®^ concentrations, a tendency of time- and concentration-dependence was observed, especially in the MPO assay (**Figures 2D–F**).

### Alveofact^®^

**Figure 3:**
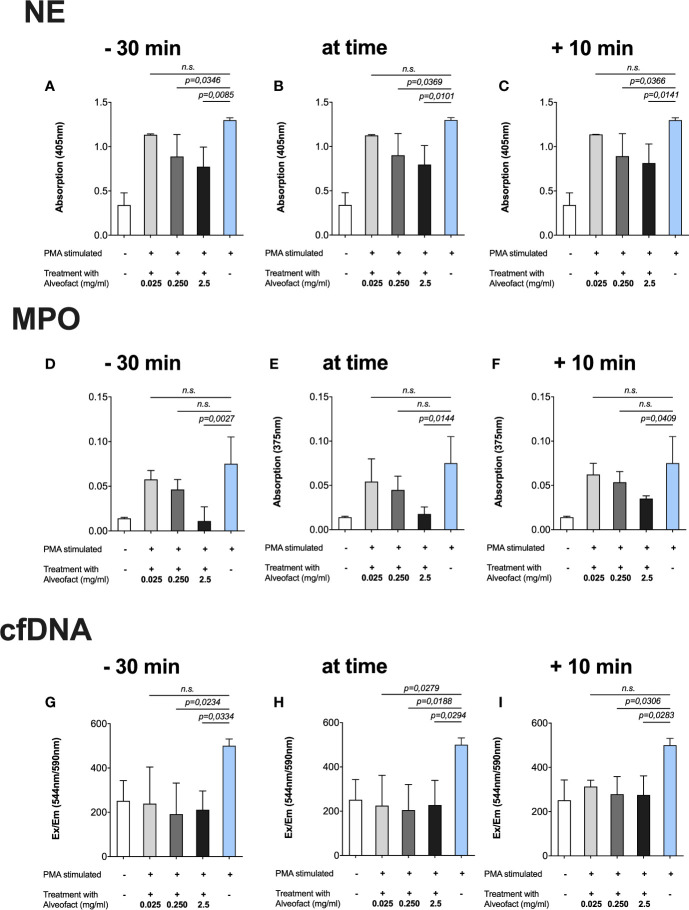
Alveofact^®^ reduces extracellular NE, MPO, and cfDNA. Neutrophils were incubated with Alveofact^®^ at three different time points in relation to activation with 100 nm PMA, and then afterwards the NET-specific components were measured in three different assays. Cells that were untreated but stimulated with 100 nm PMA served as positive control in each assay. **(A**–**C)** Concentrations of 250 µg/ml and 2.5 mg/ml could reduce the amounts of NE significantly at all time points. **(D**–**F)** The amounts of MPO were approximately as low as that in the untreated control when 2.5 mg/ml Alveofact^®^ was applied at 30 min prior to simultaneous activation with PMA. **(G**–**I)** The levels of cfDNA were about as high as that in the untreated and unstimulated control, regardless of timing and concentration of Alveofact^®^.

Even more evident results were obtained after treating the cells with Alveofact^®^ at the same time points described above. The 30 min incubation with the highest concentration (2.5 mg/ml) prior to activation with PMA generated the lowest measurements of NE (**Figure 3A**), MPO (**Figure 3D**), and cfDNA (**Figure 3G**). In simultaneous treatment with activation and treatment 10 min after activation, the highest concentration (2.5 mg/ml) also resulted in a significant reduction of NE (**Figures 3B, C**), MPO (**Figures 3E, F**), and cfDNA (**Figures 3H, I**). Unlike Curosurf^®^, the intermediate dose of 250 µg/ml Alveofact^®^ significantly reduced the amount of NE (**Figures 3A–C**) and cfDNA (**Figures 3G, H**), but not MPO (**Figures 3E, F**), at all timepoints, indicating a stronger effect of Alveofact^®^. The lowest concentration of 25 µg/ml generally had no significant effect on any of the measured factors, with one exception in the cfDNA assay for simultaneous treatment and activation (**Figure 3H**).

### Immunostained Microscopy Images Confirm the Inhibition of NETosis by Curosurf^®^ and Alveofact^®^

**Figure 4:**
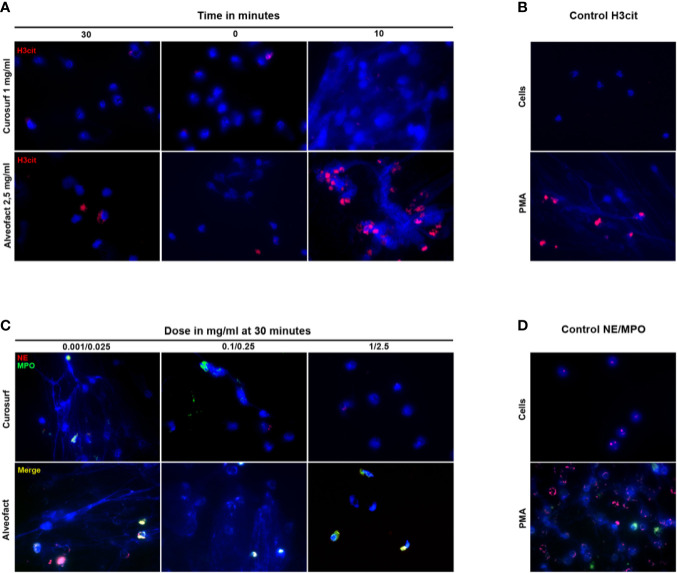
Immunostaining confirms the time- and dose-dependent suppression of NETosis. **(A)** Neutrophils were activated with PMA 30 min before, simultaneously, or 10 min after treatment with the highest dose of Curosurf^®^ (1 mg/ml) or Alveofact^®^ (2.5 mg/ml), and immunostained for H3cit (red) and for DNA with DAPI (blue). Treatment 10 min after stimulation showed no suppression of NETosis and a comparable picture to the positive control **(B)**, while the 0- and 30-min time points showed suppressed NETosis. **(B)** Negative control with untreated and unstimulated cells had no sign of NETosis. PMA-stimulated cells as positive control showed H3cit formation. **(C)** Neutrophils were treated with three different concentrations of Curosurf^®^ (0.001, 0.1, and 1 mg/ml) and Alveofact^®^ (0.025, 0.25 and 2.5 mg/ml) 30 min before activation with PMA and were immunostained for NE (red) and MPO (green), and stained for DNA with DAPI (blue). The suppression of NETosis was strongest in samples with the highest dose of surfactant and was barely or not detectable at the lower doses. **(D)** Negative controls for NE/MPO showed intact untreated and unstimulated cells with only slight NE signal; positive controls with PMA-stimulated cells showed massive NET formation with explicit NE and MPO signals co-localized to NET DNA (×40 magnification).

We used immunofluorescence staining to visualize and verify the suppression of NETosis by Curosurf^®^ or Alveofact^®^. To detect NETs, neutrophils were treated with either Curosurf^®^ or Alveofact^®^ and stimulated with PMA. Untreated and unstimulated neutrophils were used as the negative control, showing a DNA signal (blue) in the nucleus (**Figures 4B, D**). On the contrary, neutrophils incubated with PMA alone showed the common net-like DNA structure concomitant to NE (red), MPO (green), and H3cit (red) (**Figures 4B, D**). Concordantly with the results reported above, immunofluorescence staining showed decreased NE and MPO (**Figure 4C**) and H3cit (**Figure 4A**) signals with treatment involving the highest concentration of Curosurf^®^ (1 mg/ml) and Alveofact^®^ (2.5 mg/ml) 30 min prior to PMA incubation. Even under simultaneous activation with PMA, the highest dose nevertheless achieved a definite reduction of the H3cit (**Figure 4A**), NE, and MPO signals (data not shown). In contrast, the lower concentrations of both surfactant solutions exerted no relevant inhibitory effect on the neutrophils, as exemplified for NE and MPO (**Figure 4C**), and seemed to be time-independent. Thus, the microscopy images confirmed the assay findings.

### Downregulation of the P2Y6 Receptor Expression and Decreased Calcium Mobilization Without Change of Intracellular PAD4 Levels

**Figure 5:**
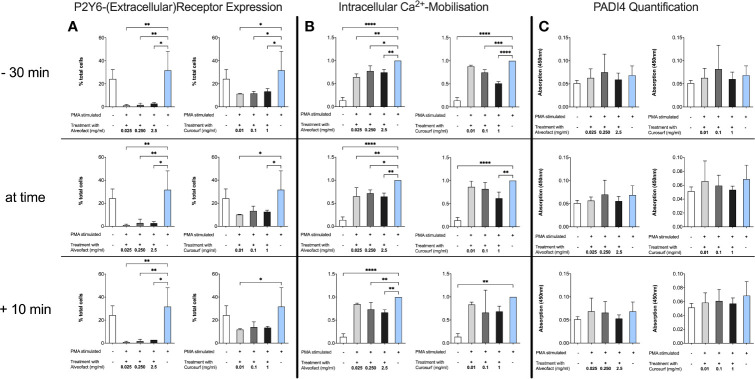
Decrease of P2Y6 Receptor and Calcium mobilization after treatment without change in intracellular PAD4 levels. **(A)** Neutrophils were treated as described before. P2Y6 was measured by flow cytometry with a FITC-conjugated antibody. Treatment with Alveofact^®^ decreases extracellular expression of the P2Y6 receptor independent of timing or dosing, while Curosurf^®^ does not exhibit this effect in every dosing. **(B)** Calcium levels at 3 h after treatment were used. Data were normalized to stimulated PMA control due to the heterogeneity in basic calcium levels and the response of the neutrophils to stimulation. Results are similar to the findings of the P2Y6 receptor expression. **(C)** Intracellular PAD4 levels were determined by ELISA using cell lysates of 1×10^6^ cells. Interestingly, PAD4 levels did not change after treatment with surfactant compared to PMA-stimulated neutrophils. Significance level was set as p<0.05 (*<0.05, **<0.005,***<0.0005, ****<0.0001).

P2Y6 receptor was investigated by Flow Cytometry using a P2Y6-FITC antibody. Neutrophils were used as control and showed the same expression patterns of the receptor as PMA stimulated neutrophils. Surfactant-treated cells showed a significantly decreased expression of the extracellular receptor (**Figure 5A**). Accordingly, to the findings of decreased receptor detection, calcium mobilization was markedly reduced compared to stimulated controls (**Figure 5B**). All findings were independent of timing or dosing for Alveofact^®^. For Curosurf^®^, a significant decrease in calcium mobilization in all concentrations was observed only at 30 min pretreatment. Interestingly PAD4 levels did not change for time or concentration (**Figure 5C**).

## Discussion

Commercial surfactant preparations consist of the lipophilic components of animal surfactants (30) but lack SP-A and SP-D. It is assumed that the primary immunoregulatory effect of surfactant is exerted by these proteins. Recent studies have shown that phospholipids and the hydrophobic proteins SP-B and SP-C, which are essential for biophysical functions, also have anti-inflammatory and antibacterial functions. They may mediate the shown effects (26–29, 34).

Our study finds that the two natural surfactants, Curosurf^®^ and Alveofact^®^, have a time- and dose-dependent inhibitory effect on NETosis *in vitro* when neutrophils have been stimulated with PMA. The stronger inhibitory effect in samples that were treated before activation with PMA implies a rather preventive than therapeutic effect on NETosis. The results suggest that the tested preparations reduce the activation of neutrophil granulocytes. Other immunologic cells like macrophages might be affected as well (20).

Regarding the underlying mechanisms of these anti-inflammatory properties, SP-B and SP-C have been demonstrated to reduce LPS-induced inflammation (35, 36). A potential mechanism of action may be based on the capability of SP-C to bind LPS *via* its N-terminal region (24). A novel synthetic surfactant, CHF5633, which contains SP-B and SP-C analogs showed to reduce LPS-induced tumor necrosis factor (TNF)-α and IL-1β cytokine production in human neonatal monocytes (37).

Several studies have investigated the potential effects of the surfactant components in transgenic knockout mice. SP-B deficiency leads to reversible pulmonary inflammation and induces neutrophil migration to the lung (27). Mice lacking SP-C have an excessive and persistent immune response of macrophages to viral and bacterial lung infections, leading to the conclusion that SP-C is a factor that minimizes inflammation. SP-D showed to inhibit LPS-induced NETosis by opsonizing pathogens (24). Other authors (38) already described the inhibitory effect of SP-D after stimulation with PMA on NETosis. Unlike SP-A and SP-D as part of the collectin family, SP-C does not opsonize pathogens, but absent SP-C results in impaired phagocytosis by alveolar macrophages (39). We found an inhibitory effect on NETosis independently of SP-D using PMA as a neutrophil activator.

Not only the proteins in pulmonary surfactant are relevant to host defense as studies on surfactant phospholipids have found. Kuronoma et al. discovered that anionic lipids of pulmonary surfactant attenuate LPS-induced inflammatory response in alveolar macrophages *via* the toll-like receptor 4–interacting proteins MD-2 and CD14 (29). Similar experiments by the same research group have revealed that the minor surfactant phospholipid palmitoyl-oleoyl-phosphatidyl-glycerol suppressed viral-elicited secretion of IL-6 and IL-8 by bronchial epithelial cells (28). Animal models and *in vivo* studies by van Rensburg et al. and Bezerra et al. have shown that exogenous surfactant administration leads to lower levels of proinflammatory biomarkers such as TNF-α, interferon (IFN)-γ, and IL-2 in the bronchoalveolar lavage fluid of mice and children (40, 41). Concomitantly there was increased expression of the anti-inflammatory cytokines IL-10 and IL-12 (41).

Dipalmitoylphosphatidylcholine, a major component of surfactant lipids, downregulates protein kinase C (PKC) and thereby reduces oxidative burst in human monocytes (42). As PMA leads to PKC-dependent activation of NADPH (nicotinamide adenine dinucleotide phosphate) oxidase in neutrophils and subsequently to NET formation, this may be a potential mechanism for the suppression of NETosis by surfactant (3). Sil et al. hypothesized that the P2Y6 receptor, a G protein-coupled receptor that transduces its signal by intracellular calcium levels, could potentially mediate NET formation by regulating IL-8 mediated neutrophil migration (43, 44). Our results, which show a decreased intracellular calcium mobilization along with decreased extracellular P2Y6 receptor expression support this statement. P2Y6 activation leads indirectly to the activation of the store-operated Ca^2+^ entry (SOCE) mechanism, which is the main mechanism of calcium signaling in neutrophils during activation (45). Researchers speculate that the SOCE inhibitor might diminish NADPH oxidase activity and by that might reduce NET formation (46). However, our study cannot provide further information whether the receptor is blocked or other mechanisms such as down-regulation or internalization of the receptor may play a role. There is currently no indication that PMA mediates its effects *via* the P2Y6 receptor. Therefore further studies are necessary to investigate the role of the P2Y6 receptor with regard to NET formation.

According to Proquitté et al., there is no significant clinical difference between Alveofact^®^ and Curosurf^®^ in terms of immediate response after administration and long term outcome parameters of treated premature infants (17). However, we observed a stronger inhibitory effect of Alveofact^®^ on NETosis, which may be attributed to the different composition. Curosurf^®^ is prepared from minced pig lungs, while Alveofact^®^ is derived from bovine lung lavage, which is less contaminated with plasmatic and tissue residue due to its manufacturing process (47). As reported in a comparative study, Alveofact^®^ contains a larger fraction of both SP-B and SP-C compared to Curosurf^®^ (15). Another possible explanation for the differing results may be the higher concentrations used for Alveofact^®^.

It is well known that surfactant proteins are also present in extrapulmonary sites such as the skin and mucosal surfaces, which has already been substantiated in previous studies (48, 40). The accelerated skin wound healing by Alveofact^®^ was attributed to downregulated gene expression of TNF-α and IL-1β as well as decreased levels of macrophages (50).

In summary, pulmonary surfactant minimizes inflammation through diverse mechanisms targeting various cell types. Many studies that have investigated these mechanisms are based on observations of alveolar macrophages and monocytes. Here, we provide the first data on the inhibition on neutrophil extracellular trap formation exerted by the two natural surfactants Alveofact^®^ and Curosurf^®^. We found evidence that the P2Y6 receptor may play a role in the inhibition of NET formation *via* interacting in the calcium pathway. Further studies are needed to decipher the details of this pathway and the underlying molecular mechanisms.

Potential limitations to this study are the lack of synthetic surfactant or individual components of the composite formulation as controls. Nonetheless Alveofact^®^ and Curosurf^®^ could provide a preventive treatment option against the formation of NETs. As NETs are of great relevance in the pathogenesis of many inflammatory lung diseases, our study indicates new perspectives on how pulmonary surfactant may be a potential candidate for attenuating inflammation.

## Data Availability Statement

The raw data supporting the conclusions of this article will be made available by the authors, without undue reservation.

## Ethics Statement

The studies involving human participants were reviewed and approved by the medical research ethics committee of the medical chamber of Hamburg.

## Author Contributions

AS and JT conceptualized the study. AS, JT, MT, BA, LR, and JK conducted the investigation. AS, JT, MB, and JK conducted the data curation and performed the formal analysis. AS and JT wrote the original draft. AS, LPR, IK, KR, MB, JT, and JPK wrote, reviewed, edited, and revised the manuscript. All authors contributed to the article and approved the submitted version.

## Conflict of Interest

The authors declare that the research was conducted in the absence of any commercial or financial relationships that could be construed as a potential conflict of interest.

## Abbreviations

NETs, Neutrophil extracellular traps; NE, neutrophil elastase; MPO, myeloperoxidase; cfDNA, cell-free DNA; H3cit, citrullinated histone H3; SP-A, surfactant protein A; SP-B, surfactant protein B; SP-C, surfactant protein C; SP-D, surfactant protein D; PMA, phorbol-12-myristate-13-acetate; BSA, bovine serum albumin; DPBS, Dulbecco’s phosphate-buffered saline; RT, room temperature; DAPI, 4′,6-diamino-2-phenylindole; LPS, lipopolysaccharide; TNF, tumor necrosis factor; IL, interleukin; IFN, interferon; PKC, protein kinase C; CD, cluster of differentiation; NADPH, nicotinamide adenine dinucleotide phosphate; CRD, carbohydrate recognition domain; FACS, fluorescence-activated cell sorting; TMB, 3,3′,5,5′-tetramethylbenzidine; SOCE, store-operated Ca^2+^ entry.
